# Effect of Montelukast on the Symptom Severity Score of Allergic Rhinitis

**DOI:** 10.7759/cureus.7403

**Published:** 2020-03-25

**Authors:** Muhammad Shaheryar Ahmed Rajput, Asif Ali Arain, Arsalan A Rajput, Mohammad Adeel, Shehzad Ghaffar, Anwar Suahil

**Affiliations:** 1 Otolaryngology, Liaquat University of Medical and Health Sciences, Jamshoro, PAK; 2 Otolaryngology, Head and Neck Surgery, King Faisal Specialist Hospital and Research Centre, Riyadh, SAU; 3 Otolaryngology, Head and Neck Surgery, Aga Khan University Hospital, Karachi, PAK; 4 Otolaryngology, The Indus Hospital, Karachi, PAK; 5 Ophthalmology, Aga Khan University Hospital, Karachi, PAK; 6 Ear, Nose & Throat, Bradford Royal Infirmary, Bradford, GBR; 7 Otolaryngology, Shaukat Khanum Memorial Cancer Hospital and Research Centre, Lahore, PAK; 8 Head and Neck Oncology, Sheffield Teaching Hospitals NHS Foundation Trust, Sheffield, GBR

**Keywords:** allergic rhinitis, montelukast, leukotriene inhibitor, antihistamine

## Abstract

Introduction

Rhinitis is a common respiratory disorder that can be broadly defined as an inflammation of nasal mucosa. Allergic rhinitis is the most common form of chronic rhinitis characterized by one or more symptoms including sneezing, nasal itching, nasal congestion, postnasal drip, and rhinorrhea. Montelukast is an antagonist to the leukotriene receptor. It is non-sedating, dosed once daily, and has a safety profile similar in adults and children, with approval down to six months of age. The purpose of the study was to see the improvement in the severity of symptoms of the patients with allergic rhinitis treated with montelukast.

Methods

The symptom severity score for allergic rhinitis was calculated by asking the patient to evaluate the severity of individual symptoms (sneezing, nasal congestion, rhinorrhea, and postnasal drip) against the 4-point scoring scale over the last 24 hours. After explaining the scoring system to the patient, a proforma was filled before starting the treatment. A dose of 10 mg of montelukast once daily was prescribed. On the follow-up visit after four weeks of therapy, compliance was ensured and then the symptom severity score was recorded again on the proforma. The total nasal symptom severity score (TNSSS) was calculated as a sum of all four nasal symptoms. Pre- and post-treatment mean of TNSSS was compared using a t-test. P-value of less than 0.05 was considered significant.

Results

A total of 140 patients were included in the study. The mean age was 30 years. The minimum age was 15 years and the maximum age was 45 years. There were 93 males and 47 females. The difference between pre- and post-mean values of TNSSS was 5.82. Both pre- and post-mean of TNSSS were compared using the t-test, and P-value was significant, i.e., <0.005.

Conclusions

The common symptoms of allergic rhinitis evaluated in the study showed improvement in response to the treatment with montelukast. The improvement in symptom severity score was maximum in sneezing and least in rhinorrhea. In light of recent developments on neuropsychiatric adverse effects and FDA warnings, caution needs to be exercised to reserve the use of montelukast for the selected patients.

## Introduction

Rhinitis is a common respiratory disorder that can be broadly defined as an inflammation of nasal mucosa. Allergic rhinitis (AR) is the most common form of chronic rhinitis characterized by one or more symptoms including sneezing, nasal itching, nasal congestion, postnasal drip, and rhinorrhea.

AR was considered as a disorder affecting the nose and nasal passages, but the evidence indicates that it is not only localized to the upper airway but it may also represent a component of a systemic disease that frequently involves the lower respiratory tract as well. This is due to a number of relationships that exist between upper and lower airways, namely physiological, functional, and immunological. For example, both the upper and lower airways are lined by ciliated epithelium with Goblet cells, which produce mucus, and they serve to clear, humidify, and warm the incoming air as it travels through the air passages [1]⁠.

Evidence has shown that allergen provocation of the upper airways leads to not only a local inflammatory response but also inflammatory processes in the lower airways. This is supported by the fact that rhinitis and asthma frequently coexist [2]⁠.

Worldwide, AR affects between 10% and 30% of the population, and sensitization (IgE [immunoglobulin E] antibodies) to foreign proteins in the environment is present in up to 40% of the population [3]. Many believe this to be a conservative estimate with about one-third of persons burdened with AR not seeking formal medical care [4]⁠.

Symptoms may have a huge adverse influence on quality of life due to frequent interference with sleep and poor work or school performance. When approaching a patient with AR symptoms, one must distinguish AR from non-AR secondary to mechanical factors such as a septal spur. The diagnosis of AR is made clinically with history and examination, and objective testing is used for a selected group of patients. Symptomatic and topical anti-inflammatory medication is often not fully effective. Autoimmune therapy can be inconvenient and expensive [5].

Montelukast is an antagonist to the leukotriene receptor. It is non-sedating, dosed once daily, and has a safety profile similar in adults and children, with approval down to six months of age. A review of the literature establishes montelukast as a viable alternative for the treatment of AR. Its benefit is equivalent to antihistamines when used as monotherapy, but less than intranasal corticosteroids [5,6]. The purpose of this study was to see the improvement in the severity of symptoms of patients with AR treated with montelukast.

## Materials and methods

The symptom severity score for AR was calculated by asking the patient to evaluate the severity of individual symptoms (sneezing, nasal congestion, rhinorrhea, and postnasal drip) against the 4-point scoring scale over the last 24 hours, as shown in Table 1.

**Table 1:** Symptom severity score

Score	Severity	Description
0	None	No symptoms
1	Mild	Symptoms occur once or twice in the last 24 hours
2	Moderate	Symptoms occur once or twice every 2 to 3 hours in a day
3	Severe	Symptoms occur every hour in a day

The inclusion criteria included the following: (1) age 15 to 45 years, (2) history and physical examination consistent with a diagnosis of AR, and (3) symptom severity score of 6 to 12. The exclusion criteria included the following: (1) patients with polyps in the nose or a significantly displaced septum, (2) patients on chronic anti-asthma medications, (3) patients treated with systemic steroids during the last 30 days, (4) physical signs or symptoms suggestive of renal, hepatic, or cardiovascular disease, (5) lactating mothers, and (6) non-compliance.

The patients visiting the outpatient department who met the inclusion criteria were enrolled for this study. Informed consent was taken from each patient after explaining the purpose and procedure of the study. A printed proforma was used to register the name, age, sex, address, and contact number of the patient. After explaining the scoring system to the patient, the proforma was filled before starting the treatment. The dose of 10-mg montelukast once daily was prescribed. On follow-up visit after four weeks of therapy, compliance was ensured and then the symptom severity score was recorded again on the proforma.

Data were entered into SPSS (Statistical Package for Social Sciences, version 16.0, SPSS Inc., Chicago, IL) and manually verified for the data entry errors. The same software was used to analyze the data. Descriptive frequencies were calculated for gender. Mean was calculated for the age and symptom severity scores of individual symptoms. The total nasal symptom severity score (TNSSS) was calculated as a sum of all four nasal symptoms. Pre- and post-treatment mean of TNSSS was compared using a t-test. P-value of less than 0.05 was considered significant.

## Results

A total of 140 patients were included in the study. The mean age was 30 years. The minimum age was 15 years and the maximum age was 45 years. There were 93 males and 47 females. The age and gender variables are shown in Table 2.

**Table 2:** Age and gender

Variables		Results
Gender	Male	93
	Female	47
Mean age		30 years
Age range		15-45 years

The mean values of pre-treatment scores for individual symptoms including sneezing, nasal obstruction, postnasal drip, and rhinorrhea were 2.38, 2.07, 2.19, and 1.61, respectively. The mean values of post-treatment scores for individual symptoms including sneezing, nasal obstruction, postnasal drip, and rhinorrhea were 0.74, 0.51, 0.71, and 0.47, respectively. The mean values of the difference between pre- and post-treatment scores for sneezing, nasal obstruction, postnasal drip, and rhinorrhea were 1.63, 1.56, 1.48, and 1.13, respectively (Table 3).

**Table 3:** Mean scores of individual nasal symptoms and differences before and after the treatment with montelukast

Nasal Symptoms	Pre-Treatment Mean Score	Post-Treatment Mean Score	Difference
Sneezing	2.38	0.74	1.63
Nasal obstruction	2.07	0.51	1.56
Post nasal drip	2.19	0.71	1.48
Rhinorrhea	1.61	0.47	1.13

The mean TNSSS before the treatment was 8.25, which reduced to 2.43 after four weeks of treatment. The difference between pre- and post-mean values of TNSSS was 5.82. Both pre- and post-treatment means of TNSSS were compared using the t-test, and the P-value was found significant, i.e., <0.005, as shown in Table 4.

**Table 4:** Pre- and post-treatment TNSSS compared using the t-test TNSSS, total nasal symptom severity score

	Pre-Treatment	Post-Treatment	P-Value
TNSSS mean	8.25	2.43	<0.005

## Discussion

Leukotrienes act as lipid mediators and are produced by cells of inflammation, such as mast cells and basophils, during the early phase of inflammatory response. Eosinophils and macrophages are responsible for the production of leukotrienes in the late phase of inflammation [7]. C4, D4, and E4 are cysteinyl leukotrienes that play a role in the development of allergic reactions in the upper and lower airways. They are derivatives of arachidonic acid through the 5-lipoxygenase pathway.

Patients with symptomatic AR have shown elevated levels of cysteinyl leukotrienes [8]. The effects of montelukast are on the function of cysteinyl leukotriene receptor 1, which is found in inflammatory cells, smooth muscle cells, and endothelial cells in the mucosa of both the upper and the lower respiratory tracts.

Montelukast is prescribed as a tablet of 10 mg in adults 15 years or older, 5 mg in children aged 6-14 years, and 4 mg in children aged 2-5 years. It is available as granules for administration to young children; the granules dissolve quickly in a glass of water. The pharmacokinetic profile allows for it to be taken once a day [9].

AR is an allergen-mediated, upper-airway inflammatory condition characterized by hyperactive respiratory mucosa, resulting in symptoms of excessive nasal secretions, sneezing, nasal itching, and congestion, with accompanying symptoms of red, itchy, watery eyes, itching of the throat and palate, and cough. AR is widely accepted as being the most common allergic disorder, but detailed statistics of its actual prevalence are lacking. Young adults are commonly affected by AR, and, consequently, it imposes a huge economic burden. The impaired quality of life due to symptoms of AR is a result of poor sleep and interruption of daily activities. AR also increases the severity of associated asthma. When montelukast was compared with placebo, the results showed improvement in disease-specific quality of life of patients with AR [10].

A randomized, placebo-controlled, 32-week study on patients with AR without the associated respiratory disease compared antihistamine treatment alone or in combination with montelukast. The results showed a gradual increase in nasal symptom improvement within six weeks of therapy with montelukast alone or in combination with an antihistamine in patients with AR [11]. Similar results have been shown in patients with AR in another study [12].

Antihistamines showed a rapid onset of action than montelukast in a study on ragweed-sensitized patients [13]. Montelukast alone improves symptoms of AR when compared with placebo [14].

Recently the U.S. Food and Drug Administration (FDA) issued warnings about serious behavior and mood-related changes with montelukast. Serious behavior and mood-related changes have been identified with the use of montelukast in observational studies. On the basis of the findings, the risks of montelukast may outweigh the benefits in some patients, particularly when the severity of the disease is mild and can be managed with available alternative therapies [15,16].

For AR, in particular, the FDA says montelukast should be reserved for patients who have not responded adequately to other therapies or who cannot tolerate these therapies [17].

## Conclusions

The common symptoms of AR evaluated in the study showed improvement in response to the treatment with montelukast. The improvement in the symptom severity score was maximum in sneezing and least in rhinorrhea. In light of recent developments on neuropsychiatric adverse effects and FDA warnings, caution needs to be exercised to reserve its use for the selected patients.
